# Supplementary material for: Seroprevalence of SARS-CoV-2 infection and associated factors among Bangladeshi slum and non-slum dwellers in pre-COVID-19 vaccination era: October 2020 to February 2021
Source: PLoS One. 2022 May 23;17(5):e0268093. doi: 10.1371/journal.pone.0268093 (PMC9126397; doi:10.1371/journal.pone.0268093)
Supplement: S1 Table — (DOCX) [file pone.0268093.s001.docx]

**Supplementary materials for**

**Seroprevalence of SARS-CoV-2 infection and associated factors among Bangladeshi slum and non-slum dwellers in pre-COVID-19 vaccination era: October 2020 to February 2021**

Rubhana Raqib^a^†, Protim Sarker^a^, Evana Akhtar^a^, Tarique Mohammad Nurul Huda^a^, Md. Ahsanul Haq^a^, Anjan Kumar Roy^a^, Md. Biplob Hosen^a^, Farjana Haque^a^, Md. Razib Chowdhury^b^, Daniel D. Reidpath^b^, Dewan Md. Emdadul Hoque^c^, Zahirul Islam^d^, Shehlina Ahmed^e^, Tahmeed Ahmed^f^, Fahmida Tofail^f^, Abdur Razzaque^b^

^a^Infectious Diseases Division, icddrb, Dhaka-1212, Bangladesh; ^b^Health Systems and Population Studies Division, icddrb, Dhaka-1212, Bangladesh; ^c^United Nations Population Fund (UNFPA) Bangladesh; ^d^Embassy of Sweden in Bangladesh; ^e^Foreign, Commonwealth & Development Office (FCDO) in Bangladesh; ^f^Nutrition and Clinical Services Division, icddrb, Dhaka-1212, Bangladesh.

†**Corresponding author:**

Rubhana Raqib

Infectious Diseases Division, icddr,b,

68 Shaheed Tajuddin Ahmed Sarani, Mohakhali, Dhaka-1212, Bangladesh

Phone: +880-2-9827068, Fax: +880-28812529

Email: [rubhana@icddrb.org](mailto:rubhana@icddrb.org)

**Short running title**: Seroprevalence of SARS-CoV-2 and associated factors

**S1 Table.** Internal Validation of the Elecsys Anti-SARS-CoV-2 Immunoassay Kit

| Measure | Estimate | 95% CI | AUC (95% CI) |
| --- | --- | --- | --- |
| Sensitivity for 7-<14 days (N=30) | 73.3% | 54.1%-87.7% | 0.87(0.79-0.95) |
| Sensitivity for 14-<21 days (N=30) | 90.0% | 73.5%-97.9% | 0.95(0.90- 1.0) |
| Sensitivity for >21 days  (N=100) | 95.0% | 88.7%-98.4% | 0.98(0.95- 1.0) |
| Total Sensitivity  (n=160) | 90.0% | 84.3%-94.2% | 0.95(0.93- 0.97) |
| Overall sensitivity, for ≥14days (n=130) | 93.8% | 88.2%-97.3% | 0.97(0.95- 0.99) |
| Overall Specificity (n=146) | 100% | 97.9%-100% | 0.97(0.95- 1.0) |
| Cross-reactivity with Common cold panel (n=51) | 0% | 93.0%-100% | 0.95(0.93-0.97) |
| Cross-reactivity with other viruses (Hepatitis B & C virus) (n=13) | 0% | 75.3%- 100% | 0.95(0.93- 97.4) |

Note: AUC: Area under curve; 95% CI: 95% Confidence interval in brackets
