# Supplementary material for: Seroprevalence of SARS-CoV-2 infection and associated factors among Bangladeshi slum and non-slum dwellers in pre-COVID-19 vaccination era: October 2020 to February 2021
Source: PLoS One. 2022 May 23;17(5):e0268093. doi: 10.1371/journal.pone.0268093 (PMC9126397; doi:10.1371/journal.pone.0268093)
Supplement: S2 Table — (DOCX) [file pone.0268093.s002.docx]

**Supplementary materials for**

**Seroprevalence of SARS-CoV-2 infection and associated factors among Bangladeshi slum and non-slum dwellers in pre-COVID-19 vaccination era: October 2020 to February 2021**

Rubhana Raqib^a^†, Protim Sarker^a^, Evana Akhtar^a^, Tarique Mohammad Nurul Huda^a^, Md. Ahsanul Haq^a^, Anjan Kumar Roy^a^, Md. Biplob Hosen^a^, Farjana Haque^a^, Md. Razib Chowdhury^b^, Daniel D. Reidpath^b^, Dewan Md. Emdadul Hoque^c^, Zahirul Islam^d^, Shehlina Ahmed^e^, Tahmeed Ahmed^f^, Fahmida Tofail^f^, Abdur Razzaque^b^

^a^Infectious Diseases Division, icddrb, Dhaka-1212, Bangladesh; ^b^Health Systems and Population Studies Division, icddrb, Dhaka-1212, Bangladesh; ^c^United Nations Population Fund (UNFPA) Bangladesh; ^d^Embassy of Sweden in Bangladesh; ^e^Foreign, Commonwealth & Development Office (FCDO) in Bangladesh; ^f^Nutrition and Clinical Services Division, icddrb, Dhaka-1212, Bangladesh.

†**Corresponding author:**

Rubhana Raqib

Infectious Diseases Division, icddr,b,

68 Shaheed Tajuddin Ahmed Sarani, Mohakhali, Dhaka-1212, Bangladesh

Phone: +880-2-9827068, Fax: +880-28812529

Email: [rubhana@icddrb.org](mailto:rubhana@icddrb.org)

**Short running title**: Seroprevalence of SARS-CoV-2 and associated factors

**S2 Table.** Weighted Seroprevalence of SARS-CoV-2 antibodies among the residents of slum and non-slum neighborhoods of the Dhaka and Chattogram districts.

|  | Dhaka (n=2614) | | | Chattogram (n=606) | | |
| --- | --- | --- | --- | --- | --- | --- |
| Variables | Overall (n=2614) | Slum (n=1910) | Non-slum (n=705) | Overall (n=606) | Slum (334) | Non-slum (272) |
|  | Prevalence (95% CI) | Prevalence (95% CI) | Prevalence (95% CI) | Prevalence (95% CI) | Prevalence (95% CI) | Prevalence (95% CI) |
| Overall | 72.9(70.6, 75.1) | 76.6(74.6, 78.6) | 67.8(43.8, 55.7) | 54.2(50.1, 58.3) | 57.7(52.1, 63.1) | 49.7(43.8, 55.7) |
| Sex |  |  |  |  |  |  |
| Male | 71.5(68.1, 74.8) | 74.8(71.5, 77.8) | 66.5(59.1, 73.1) | 49.9(43.8, 56.0) | 50.8(42.7, 58.9) | 48.5(39.2, 57.8) |
| Female | 73.9(70.8, 76.8) | 78.1(75.4, 80.6) | 68.7(62.6, 74.2) | 57.9(52.4, 63.2) | 64.5(56.7, 71.6) | 50.6(42.8, 58.3) |
| Age, distribution |  |  |  |  |  |  |
| 10-14 years | 69.7(63.4, 75.4) | 73.3(67.8, 78.2) | 62.6(47.4, 75.7) | 44.2(35.5, 53.3) | 44.3(33.3, 55.8) | 44.1(30.0, 59.3) |
| 15-17 years | 71.9(63.0, 79.4) | 78.2(70.2, 84.5) | 63.5(45.9, 78.1) | 66.2(53.0, 77.4) | 73.2(57.0, 84.9) | 50.3(26.6, 73.8) |
| 18-30 years | 73.0(68.8, 76.9) | 76.1(72.1, 80.0) | 69.1(60.7, 76.3) | 55.1(47.2, 62.7) | 63.3(52.7, 72.7) | 43.1(31.9, 55.1) |
| 31-50 years | 73.9(70.0, 77.6) | 78.0(74.2, 81.3) | 68.5(60.4, 75.7) | 58.2(50.5, 65.5) | 61.9(50.6, 72.1) | 54.5(43.9, 64.8) |
| >50 years | 74.2(68.0, 79.5) | 76.8 (71.2, 81.6) | 70.9 (58.9, 80.) | 53.3(42.4, 63.9) | 49.4(31.5, 67.4) | 55.5(42.1, 68.2) |
| Years of education |  |  |  |  |  |  |
| No education | 71.7(67.6, 75.5) | 74.9(71.4, 78.1) | 57.5(42.6, 71.1) | 50.4(39.0, 61.9) | 55.3(42.1, 67.8) | 32.7(14.8, 57.5) |
| 1-5 years | 77.0(73.4, 80.3) | 78.2(74.8, 81.2) | 73.3(61.9, 82.2) | 53.8(45.6, 61.9) | 55.3(45.9, 64.4) | 47.7(31.3, 64.6) |
| 6-10 years | 72.5(67.6, 76.9) | 76.6(71.8, 80.8) | 69.8(62.4, 76.3) | 55.2(48.7, 61.4) | 59.7(50.7, 68.0) | 50.0(40.7, 59.1) |
| 11-15 years | 67.7(60.3, 74.3) | 80.3(66.2, 89.4) | 66.3(58.2, 73.5) | 55.6(46.9, 63.9) | 70.0(47.0, 85.8) | 52.9(43.6, 62.1) |
| Occupation |  |  |  |  |  |  |
| Service | 74.8(68.8, 80.0) | 80.1(74.7, 84.6) | 67.7(55.7, 77.8) | 67.0(55.8, 76.5) | 75.2(61.0, 85.5) | 56.3(39.3, 71.9) |
| Self employed | 71.0(63.7, 77.3) | 74.2(68.0, 79.5) | 58.6(35.0, 78.0) | 54.9(42.4, 66.8) | 55.5(42.1, 68.1) | 50.0(20.0, 80.2) |
| Business | 70.0(61.7, 77.1) | 73.8(65.4, 80.7) | 65.3(50.0, 78.1) | 54.0(38.0, 69.2) | 69.2(40.8, 88.0) | 45.5(27.1, 65.2) |
| Homemaker | 76.8(72.3, 80.8) | 82.0(78.0, 85.5) | 71.8(63.7, 78.7) | 56.4(48.6, 63.8) | 64.4(52.8, 74.5) | 50.0(39.2, 60.0) |
| Unemployed | 68.5(62.9, 73.7) | 70.9(65.9, 75.5) | 62.5(47.2, 75.7) | 48.5(34.5, 62.6) | 44.5(25.0, 65.8) | 52.1(33.7, 69.9) |
| Student | 72.2(67.2, 76.7) | 76.7(72.0, 80.8) | 67.7(58.6, 75.5) | 48.9(41.9, 55.9) | 50.0(40.4, 59.2) | 47.5(37.3, 57.9) |
| BMI |  |  |  |  |  |  |
| Normal | 71.5(67.9, 74.8) | 76.5(73.4, 79.3) | 64.1(56.8, 70.9) | 51.7(45.1, 58.3) | 59.1(50.2, 67.4) | 40.7(31.3, 50.8) |
| Underweight | 71.1(65.4, 76.1) | 72.6(67.7, 77.0) | 67.7(52.8, 79.8) | 42.5(34.6, 50.8) | 44.1(35.0, 53.5) | 36.7(22.2, 54.1) |
| Overweight | 75.6(71.9, 78.9) | 79.5(75.9, 82.6) | 71.5(65.0, 77.3) | 65.7(59.2, 71.7) | 77.2(66.7, 85.2) | 59.2(50.9, 67.0) |

Data has been presented as prevalence with 95% CI.
