# Supplementary material for: Seroprevalence of SARS-CoV-2 infection and associated factors among Bangladeshi slum and non-slum dwellers in pre-COVID-19 vaccination era: October 2020 to February 2021
Source: PLoS One. 2022 May 23;17(5):e0268093. doi: 10.1371/journal.pone.0268093 (PMC9126397; doi:10.1371/journal.pone.0268093)
Supplement: S3 Table — (DOCX) [file pone.0268093.s003.docx]

**Supplementary materials for**

**Seroprevalence of SARS-CoV-2 infection and associated factors among Bangladeshi slum and non-slum dwellers in pre-COVID-19 vaccination era: October 2020 to February 2021**

Rubhana Raqib^a^†, Protim Sarker^a^, Evana Akhtar^a^, Tarique Mohammad Nurul Huda^a^, Md. Ahsanul Haq^a^, Anjan Kumar Roy^a^, Md. Biplob Hosen^a^, Farjana Haque^a^, Md. Razib Chowdhury^b^, Daniel D. Reidpath^b^, Dewan Md. Emdadul Hoque^c^, Zahirul Islam^d^, Shehlina Ahmed^e^, Tahmeed Ahmed^f^, Fahmida Tofail^f^, Abdur Razzaque^b^

^a^Infectious Diseases Division, icddrb, Dhaka-1212, Bangladesh; ^b^Health Systems and Population Studies Division, icddrb, Dhaka-1212, Bangladesh; ^c^United Nations Population Fund (UNFPA) Bangladesh; ^d^Embassy of Sweden in Bangladesh; ^e^Foreign, Commonwealth & Development Office (FCDO) in Bangladesh; ^f^Nutrition and Clinical Services Division, icddrb, Dhaka-1212, Bangladesh.

†**Corresponding author:**

Rubhana Raqib

Infectious Diseases Division, icddr,b,

68 Shaheed Tajuddin Ahmed Sarani, Mohakhali, Dhaka-1212, Bangladesh

Phone: +880-2-9827068, Fax: +880-28812529

Email: [rubhana@icddrb.org](mailto:rubhana@icddrb.org)

**Short running title**: Seroprevalence of SARS-CoV-2 and associated factors

**S3 Table.** Weighted seroprevalence of SARS-CoV-2 antibodies among the participants with self-reported occurrence of COVID-like symptoms in the past six months.

| Variables | Overall (n=1145) | Slum  (n=671) | Non-slum  (n=474) |
| --- | --- | --- | --- |
| Symptoms | Prevalence (95% CI) | Prevalence (95% CI) | Prevalence (95% CI) |
| Fever |  |  |  |
| Presence | 70.9(67.4, 74.2) | 70.7(66.4, 74.6) | 71.2(65.3, 76.4) |
| Absence | 53.1(37.6, 68.0) | 54.5(26.7, 80.0) | 52.8(34.9, 70.0) |
| Dry cough |  |  |  |
| Presence | 72.8(68.9, 76.5) | 76.4(72.3, 80.1) | 67.0(58.8, 74.3) |
| Absence | 64.7(58.3, 70.5) | 53.0(42.8, 63.0) | 71.1(63.1, 78.0) |
| Sore throat |  |  |  |
| Presence | 75.8(69.3, 81.3) | 76.1(67.8, 82.8) | 75.6(65.5, 83.5) |
| Absence | 67.6(63.6, 71.5) | 68.7(63.8, 73.2) | 66.4(59.5, 72.7) |
| Diarrhea |  |  |  |
| Presence | 70.0(53.8, 82.4) | 70.6(45.4, 87.4) | 69.3(48.2, 84.6) |
| Absence | 69.7(66.2, 73.0) | 70.3(66.0, 74.2) | 69.1(63.2, 74.4) |
| Loss of taste or smell |  |  |  |
| Presence | 72.6(65.1, 79.0) | 69.8(60.1, 78.0) | 76.0(63.6, 85.2) |
| Absence | 69.0(65.1, 72.6) | 70.4(65.7, 74.7) | 67.5(61.0, 73.3) |
| Reported any 3 symptoms |  |  |  |
| Presence | 76.6(70.3, 81.9) | 75.0(66.4, 82.0) | 78.2(68.3, 85.7) |
| Absence | 67.4(63.3, 71.2) | 68.7(63.8, 73.2) | 65.9(59.1, 72.2) |

Data has been presented as prevalence with 95% CI in brackets.
