# Supplementary material for: Seroprevalence of SARS-CoV-2 infection and associated factors among Bangladeshi slum and non-slum dwellers in pre-COVID-19 vaccination era: October 2020 to February 2021
Source: PLoS One. 2022 May 23;17(5):e0268093. doi: 10.1371/journal.pone.0268093 (PMC9126397; doi:10.1371/journal.pone.0268093)
Supplement: S4 Table — (DOCX) [file pone.0268093.s004.docx]

**Supplementary materials for**

**Seroprevalence of SARS-CoV-2 infection and associated factors among Bangladeshi slum and non-slum dwellers in pre-COVID-19 vaccination era: October 2020 to February 2021**

Rubhana Raqib^a^†, Protim Sarker^a^, Evana Akhtar^a^, Tarique Mohammad Nurul Huda^a^, Md. Ahsanul Haq^a^, Anjan Kumar Roy^a^, Md. Biplob Hosen^a^, Farjana Haque^a^, Md. Razib Chowdhury^b^, Daniel D. Reidpath^b^, Dewan Md. Emdadul Hoque^c^, Zahirul Islam^d^, Shehlina Ahmed^e^, Tahmeed Ahmed^f^, Fahmida Tofail^f^, Abdur Razzaque^b^

^a^Infectious Diseases Division, icddrb, Dhaka-1212, Bangladesh; ^b^Health Systems and Population Studies Division, icddrb, Dhaka-1212, Bangladesh; ^c^United Nations Population Fund (UNFPA) Bangladesh; ^d^Embassy of Sweden in Bangladesh; ^e^Foreign, Commonwealth & Development Office (FCDO) in Bangladesh; ^f^Nutrition and Clinical Services Division, icddrb, Dhaka-1212, Bangladesh.

†**Corresponding author:**

Rubhana Raqib

Infectious Diseases Division, icddr,b,

68 Shaheed Tajuddin Ahmed Sarani, Mohakhali, Dhaka-1212, Bangladesh

Phone: +880-2-9827068, Fax: +880-28812529

Email: [rubhana@icddrb.org](mailto:rubhana@icddrb.org)

**Short running title**: Seroprevalence of SARS-CoV-2 and associated factors

**S4 Table.** Weighted seroprevalence of SARS-CoV-2 antibodies among the participants with co-morbidities.

| Variables | Overall (n=2444) | Slum  (n=1564) | Non-slum  (n=880) |
| --- | --- | --- | --- |
| Comorbidities (Adults) | Prevalence (95% CI) | Prevalence (95% CI) | Prevalence (95% CI) |
| Diabetes |  |  |  |
| Presence | 77.5(71.1, 82.9) | 81.5(72.6, 88.1) | 75.8(67.2, 82.7) |
| Absence | 67.3(64.8, 69.8) | 72.3(69.6, 74.9) | 60.2(55.5, 64.8) |
| Stroke |  |  |  |
| Presence | 77.4(65.8, 85.9) | 74.3(58.3, 85.7) | 62.9(58.6, 67.0) |
| Absence | 68.5(66.1, 70.8) | 73.0(70.4, 75.5) | 80.1(62.5, 90.7) |
| Heart disease |  |  |  |
| Presence | 74.1(63.9, 82.2) | 80.3(68.0, 88.6) | 68.3(51.7, 81.2) |
| Absence | 68.5(66.1, 70.8) | 72.7(70.0, 75.2) | 63.3(59.0, 67.4) |
| Hypertension |  |  |  |
| Presence | 71.5(66.4, 76.2) | 78.9(72.8, 84.0) | 65.5(57.4, 72.7) |
| Absence | 68.1(65.4, 70.6) | 71.8(68.9, 74.6) | 63.0(58.1, 67.6) |
| Lung diseases |  |  |  |
| Presence | 66.0(49.1, 79.2) | 76.1(54.6, 89.4) | 56.3(32.1, 77.9) |
| Absence | 68.9(66.5, 71.1) | 73.0(70.4, 75.4) | 63.8(60.0, 67.9) |
| Asthma |  |  |  |
| Presence | 66.7(57.2, 75.0) | 69.4(57.0, 79.5) | 64.6(50.3, 76.7) |
| Absence | 68.9(66.5, 71.2) | 73.2(70.6, 75.7) | 63.5(59.2, 67.7) |
| Cancer |  |  |  |
| Presence | 63.4(40.0, 81.8) | 72.4(41.3, 90.7) | 54.4(23.0, 82.6) |
| Absence | 68.8(66.5, 71.1) | 73.0(70.4, 75.5) | 63.7(59.5, 67.7) |

Results was presented as prevalence with 95% confidence interval in brackets.
