# Supplementary material for: Seroprevalence of SARS-CoV-2 infection and associated factors among Bangladeshi slum and non-slum dwellers in pre-COVID-19 vaccination era: October 2020 to February 2021
Source: PLoS One. 2022 May 23;17(5):e0268093. doi: 10.1371/journal.pone.0268093 (PMC9126397; doi:10.1371/journal.pone.0268093)
Supplement: S1 File — (DOCX) [file pone.0268093.s005.docx]

**Supplementary materials for**

**Seroprevalence of SARS-CoV-2 infection and associated factors among Bangladeshi slum and non-slum dwellers in pre-COVID-19 vaccination era: October 2020 to February 2021**

Rubhana Raqib^a^†, Protim Sarker^a^, Evana Akhtar^a^, Tarique Mohammad Nurul Huda^a^, Md. Ahsanul Haq^a^, Anjan Kumar Roy^a^, Md. Biplob Hosen^a^, Farjana Haque^a^, Md. Razib Chowdhury^b^, Daniel D. Reidpath^b^, Dewan Md. Emdadul Hoque^c^, Zahirul Islam^d^, Shehlina Ahmed^e^, Tahmeed Ahmed^f^, Fahmida Tofail^f^, Abdur Razzaque^b^

^a^Infectious Diseases Division, icddrb, Dhaka-1212, Bangladesh; ^b^Health Systems and Population Studies Division, icddrb, Dhaka-1212, Bangladesh; ^c^United Nations Population Fund (UNFPA) Bangladesh; ^d^Embassy of Sweden in Bangladesh; ^e^Foreign, Commonwealth & Development Office (FCDO) in Bangladesh; ^f^Nutrition and Clinical Services Division, icddrb, Dhaka-1212, Bangladesh.

†**Corresponding author:**

Rubhana Raqib

Infectious Diseases Division, icddr,b,

68 Shaheed Tajuddin Ahmed Sarani, Mohakhali, Dhaka-1212, Bangladesh

Phone: +880-2-9827068, Fax: +880-28812529

Email: [rubhana@icddrb.org](mailto:rubhana@icddrb.org)

**Short running title**: Seroprevalence of SARS-CoV-2 and associated factors

**S1 File.**

**Field data collection and study team:** Field work of Dhaka slums was conducted by icddr,b, while for non-slum of Dhaka and slum and non-slum areas of Chattogram, ‘Mitra and Associate’(consulting firm), was engaged for data collection.

For data collection of Dhaka slums, two weeks training program was organized by icddr,b for the field workers that includes both in office and field test. Under the guidance of PIs, Field Coordinator of UHDSS coordinated the training program, while respective trainers and field workers participated (questionnaire survey, measurements and blood specimen collection). For data collection of Dhaka non-slum and Chattogram slum and non-slum, Field Coordinator of UHDSS (icddr,b) along with his team members was involved as a trainer, to train the trainers of ‘Mitra and Associates’. Subsequently the trainers of ‘Mitra and Associates’ trained the field workers, while icddr,b team oversaw the training program. The training program continued for two weeks. The icddr,b team was also responsible for data quality checks of data (survey, measurements and blood collection) during data collection period both in Dhaka and Chattogram.

To maintain icddr,b’s safety policy, the study field team received training on COVID-19 related biosafety measures and were provided with personal protective equipment such as masks, gloves, apron, and sanitizers etc to use during field visits. The field team was given extensive training to motivate and obtain informed consent or assent from family members for participation in the study and to donate blood. During the data collection in the field, the quality checks were done by a quality assessment team.
