# Supplementary material for: Seroprevalence of SARS-CoV-2 infection and associated factors among Bangladeshi slum and non-slum dwellers in pre-COVID-19 vaccination era: October 2020 to February 2021
Source: PLoS One. 2022 May 23;17(5):e0268093. doi: 10.1371/journal.pone.0268093 (PMC9126397; doi:10.1371/journal.pone.0268093)
Supplement: S2 File — (DOCX) [file pone.0268093.s006.docx]

**Supplementary materials for**

**Seroprevalence of SARS-CoV-2 infection and associated factors among Bangladeshi slum and non-slum dwellers in pre-COVID-19 vaccination era: October 2020 to February 2021**

Rubhana Raqib^a^†, Protim Sarker^a^, Evana Akhtar^a^, Tarique Mohammad Nurul Huda^a^, Md. Ahsanul Haq^a^, Anjan Kumar Roy^a^, Md. Biplob Hosen^a^, Farjana Haque^a^, Md. Razib Chowdhury^b^, Daniel D. Reidpath^b^, Dewan Md. Emdadul Hoque^c^, Zahirul Islam^d^, Shehlina Ahmed^e^, Tahmeed Ahmed^f^, Fahmida Tofail^f^, Abdur Razzaque^b^

^a^Infectious Diseases Division, icddrb, Dhaka-1212, Bangladesh; ^b^Health Systems and Population Studies Division, icddrb, Dhaka-1212, Bangladesh; ^c^United Nations Population Fund (UNFPA) Bangladesh; ^d^Embassy of Sweden in Bangladesh; ^e^Foreign, Commonwealth & Development Office (FCDO) in Bangladesh; ^f^Nutrition and Clinical Services Division, icddrb, Dhaka-1212, Bangladesh.

†**Corresponding author:**

Rubhana Raqib

Infectious Diseases Division, icddr,b,

68 Shaheed Tajuddin Ahmed Sarani, Mohakhali, Dhaka-1212, Bangladesh

Phone: +880-2-9827068, Fax: +880-28812529

Email: [rubhana@icddrb.org](mailto:rubhana@icddrb.org)

**Short running title**: Seroprevalence of SARS-CoV-2 and associated factors

**S2 File**

**Sample size**

In the absence of available seroprevalence data in Bangladesh, we performed a pilot study to perform the sample size calculation for the larger study. The seropositivity among 300 study participants was 67% with standard error of 0.02. Using this estimate, and choosing a precision of 2% and a confidence interval of 95%, the estimated sample size was 2124. Knowing, however that the study involved cluster sampling, we used the design effect as a correction factor to adjust the required sample size to overcome within cluster/household correlations. The estimated design effect was 1.5, leading to a final sample size of 3,186 (rounded to 3200).
