# Supplementary material for: Seroprevalence of SARS-CoV-2 infection and associated factors among Bangladeshi slum and non-slum dwellers in pre-COVID-19 vaccination era: October 2020 to February 2021
Source: PLoS One. 2022 May 23;17(5):e0268093. doi: 10.1371/journal.pone.0268093 (PMC9126397; doi:10.1371/journal.pone.0268093)
Supplement: Attachment — Submitted filename: Review_Seroprevalence of SARS-CoV-2 and associated factors.doc [file pone.0268093.s007.doc]

**Review**: **Seroprevalence of SARS-CoV-2 infection and associated factors among slum and non-slum dwellers in Bangladesh during October 2020 to February 2021**

**Reviewer**: Amos Hamukale

Recommend approval. This is an interesting and important paper. Limited testing capacity has incapacitated many third world countries from knowing the true spread and impact of Covid-19. As the authors highlight, Limited population-based serosurveys for SARS-CoV-2 have been conducted in low- and medium-income countries reflecting a dearth of focused serosurveys among the potentially vulnerable populations. This study adds to the limited information on the impact that socio-economic factors have on the burden of SARS-CoV-2. Most of my comments are clarifying and I did not find any major issues with the methods/results/conclusions of this paper.

***Comments***

**Manuscript**:

Line 141-143: highlights that during lockdown accessibility to non-slum households was limited hence collected data from 2118 slum inhabitants and 1102 from non-slum in a 2:1 ratio. It is not clear how sample size was determined, it seems the sample size was adjusted to suit the non-response in households that could not be reached hence introducing systematic bias.

Line 210-211: The mean with standard deviation are reported for continuous data but there is not indication that the data was checked for normality which would determine whether to use mean or median.

Line 214-216 & 238: Authors indicated that they conducted a weighted analysis but did not provide adequate information on what kind of weights were used for example Design weights or post-Stratification weights for the different variables.

Table 1: Age in years should be categorized to better understand the distribution and how they affect the analysis. Having more children in one group can easily skew the data hence giving a false average.

Table 1: Authors have indicated p-value in line 248-249 but these are not reported in the table.

Table 3: On years of education, the authors use 11-15 years as the reference group. This reference group does not adequately represent the Slum dwellers 3% hence reduces the precision.
