# Supplementary material for: Seroprevalence of SARS-CoV-2 infection and associated factors among Bangladeshi slum and non-slum dwellers in pre-COVID-19 vaccination era: October 2020 to February 2021
Source: PLoS One. 2022 May 23;17(5):e0268093. doi: 10.1371/journal.pone.0268093 (PMC9126397; doi:10.1371/journal.pone.0268093)
Supplement: Attachment — Submitted filename: Response to Reviewers.docx [file pone.0268093.s009.docx]

**Comments from Academic Editor and Managerial Desk**

**Comment on 29 March 2022**

-We note you updated your Data Availability statement to the following:

- "Yes - all data are fully available without restriction"
- "All datasets used in this study are available."

While you have stated that the data is fully available without legal or ethical restrictions, you have not explained where the data can be found and how others may access the data.

**Response**: We have added the Data Availability statement describing the compliance with PLOS' data policy in the MS (in data availability section line number:405-409) as well as in the MS submission system. The revised manuscript was submitted on 3^rd^ April 2022.

**Comment on 13 April 2022**

-We are requesting for an updated version of your manuscript including the ethical approval number provided by the institutional review board.

**Response**: As requested, we have added the ethical approval number (PR-20070, dated 1^st^ September 2020) provided by the Ethical Review Committee (ERC) of icddr,b in the manuscript Lines 193-196. However, it was not possible to upload the revised manuscript in the submission system, as the system did not allow uploading. Therefore, we replied to the mail by attaching the revised manuscript.

**Comment on 20^th^ April 2022**

-The editorial manager system can now allow you to upload your updated revised manuscript. Therefore, we invite you to submit a revised version of the manuscript that addresses the points raised during the review process. Please include the following items when submitting your revised manuscript:

- A rebuttal letter that responds to each point raised by the academic editor and reviewer(s). You should upload this letter as a separate file labeled 'Response to Reviewers'.
- A marked-up copy of your manuscript that highlights changes made to the original version. You should upload this as a separate file labeled 'Revised Manuscript with Track Changes'.
- An unmarked version of your revised paper without tracked changes. You should upload this as a separate file labeled 'Manuscript'

**Response**: There are no further comments from the reviewers after submission of the revised manuscript and response to Reviewers’ comments on 10^th^ February 2022. The rebuttal letter that responds to point raised by the academic editor and Managerial Desk have been addressed in the file labeled as 'Response to Reviewers'.

As indicated by the Academic Editor, we have now uploaded a revised copy of the manuscript with revision marked in track changes, labeled as “Revised Manuscript with Track Changes” and a clean unmarked copy of the revised manuscript labelled as “Manuscript”.
